# Insulin Resistance in Non-Obese Subjects Is Associated with Activation of the JNK Pathway and Impaired Insulin Signaling in Skeletal Muscle

**DOI:** 10.1371/journal.pone.0019878

**Published:** 2011-05-11

**Authors:** Umesh B. Masharani, Betty A. Maddux, Xiaojuan Li, Giorgos K. Sakkas, Kathleen Mulligan, Morris Schambelan, Ira D. Goldfine, Jack F. Youngren

**Affiliations:** 1 Department of Medicine, Diabetes Center, University of California San Francisco, San Francisco, California, United States of America; 2 Department of Radiology, Musculo-Skeletal Quantitative Imaging Research (MQIR), University of California San Francisco, San Francisco, California, United States of America; 3 Division of Endocrinology, San Francisco General Hospital, San Francisco, California, United States of America; University of Tor Vergata, Italy

## Abstract

**Background:**

The pathogenesis of insulin resistance in the absence of obesity is unknown. In obesity, multiple stress kinases have been identified that impair the insulin signaling pathway via serine phosphorylation of key second messenger proteins. These stress kinases are activated through various mechanisms related to lipid oversupply locally in insulin target tissues and in various adipose depots.

**Methodology/Principal Findings:**

To explore whether specific stress kinases that have been implicated in the insulin resistance of obesity are potentially contributing to insulin resistance in non-obese individuals, twenty healthy, non-obese, normoglycemic subjects identified as insulin sensitive or resistant were studied. Vastus lateralis muscle biopsies obtained during euglycemic, hyperinsulinemic clamp were evaluated for insulin signaling and for activation of stress kinase pathways. Total and regional adipose stores and intramyocellular lipids (IMCL) were assessed by DXA, MRI and ^1^H-MRS. In muscle of resistant subjects, phosphorylation of JNK was increased (1.36±0.23 vs. 0.78±0.10 OD units, *P*<0.05), while there was no evidence for activation of p38 MAPK or IKKβ. IRS-1 serine phosphorylation was increased (1.30±0.09 vs. 0.22±0.03 OD units, *P*<0.005) while insulin-stimulated tyrosine phosphorylation decreased (10.97±0.95 vs. 0.89±0.50 OD units, *P*<0.005). IMCL levels were twice as high in insulin resistant subjects (3.26±0.48 vs. 1.58±0.35% H_2_O peak, *P*<0.05), who also displayed increased total fat and abdominal fat when compared to insulin sensitive controls.

**Conclusions:**

This is the first report demonstrating that insulin resistance in non-obese, normoglycemic subjects is associated with activation of the JNK pathway related to increased IMCL and higher total body and abdominal adipose stores. While JNK activation is consistent with a primary impact of muscle lipid accumulation on metabolic stress, further work is necessary to determine the relative contributions of the various mediators of impaired insulin signaling in this population.

## Introduction

Skeletal muscle insulin resistance contributes to the development of type 2 diabetes mellitus (T2D) and plays a causal role in cardiovascular disease and related disorders [1], [2]. The mechanisms underlying cellular resistance to insulin, however, remain unclear. Adiposity has a strong negative impact on insulin action in skeletal muscle [3]–[5], so much of the work exploring the mechanisms of insulin resistance have employed human and animal models of obesity. Results from these various studies indicate that impairments in insulin signaling are mediated in insulin target tissues by stress kinases such as c-Jun NH_2_-terminal kinase (JNK), p38 mitogen-activated protein kinase (MAPK), and inhibitor of NF-κB kinase complex beta (IKKβ). These kinases phosphorylate several key, negative regulatory, serine residues on the insulin receptor (IR) and IRS-1 molecules, most notably serine^312^ of IRS-1 (for reviews see [6], [7]), resulting in the diminished activity of enzymes in the phosphatidylinositol 3-kinase (PI3K)/Akt pathway observed in insulin resistant tissues [8], [9].

These kinases represent multiple cross-reactive cellular stress-response pathways that can be activated either as a result of local factors induced by the accumulation and/or metabolism of intracellular lipids, or by circulating adipose-secreted cytokines such as tumor necrosis factor-alpha (TNF-α) [10]–[12]. This activation of multiple stress kinases in response to an over-accumulation of lipids describes a generalized inflammatory state that, in obesity, has contributions from both an expansion of whole body adipose depots and a related cellular stress response resulting from the deposition of ectopic lipids within muscle cells [13], [14].

Insulin resistance also occurs in lean individuals, however, and the mechanisms contributing to impaired insulin signaling in the absence of obesity are much less well characterized. In a study of a specific population of insulin resistant, non-obese subjects, an increased serine phosphorylation of IRS-1 on multiple negative regulatory sites was observed in muscle biopsies from first degree off spring of parents with type 2 diabetes mellitus [15]. While these data suggest that stress kinases are involved in that population, no specific serine kinases have been implicated in insulin resistance in the absence of obesity.

Furthermore, whether the inflammatory responses to lipid accumulation are the ultimate cause of impaired insulin signaling in non-obese subjects is unknown. It has been reported that, even in subjects of normal body weight, insulin resistance is associated with greater whole body fat stores as well as higher levels of muscle lipids [16]. However, the total amount of adipose tissue as well as the intramyocellular lipid stores are much smaller in healthy non-obese than in obese individuals [17], making it unclear whether the range at which these variables occur in an otherwise healthy, non-obese population reach a putative threshold required to induce a stress response in insulin target tissues. The relationship of adiposity or insulin action to circulating levels of adipose-secreted inflammatory cytokines, such as TNF-α, have not been well studied in the absence of obesity. Thus, it is not clear whether a relatively small expansion of adipose tissue in overall lean individuals can transmit an adiposity signal to insulin target tissues [11]–[14]. Similarly, a correlation between elevated intramyocellular lipids (IMCL) with decreased insulin action has been demonstrated in non-obese subjects [17]–[19], but it has not been demonstrated that IMCL accumulation in this population is associated with activation of any stress pathways.

To identify specific stress kinase pathways that might be associated with impaired insulin signaling in the absence of obesity, and to determine the impact of lipid accumulation on these cellular signaling events, we have studied stress kinase activation in skeletal muscle biopsies from in non-obese, insulin resistant and insulin sensitive subjects with normal glucose tolerance, and quantified total and visceral fat stores, and IMCL content. These studies present the first evidence for activation of the JNK stress kinase pathway in muscle from insulin resistant, non-obese subjects, and demonstrate a corresponding increase in IRS-1 serine phosphorylation with diminished insulin signaling.

## Results

### Clinical Characteristics

Subject characteristics are shown in **Table 1**. Twenty subjects were segregated based on high or low glucose disposal values during the hyperinsulinemic clamp (>12.0 or <9.5 mg^−1^⋅min^−1^⋅kg_LBM_^−1^⋅SSI^−1^, respectively). BMI values were not significantly different between insulin sensitive (SEN) and insulin resistant (RES) groups. Likewise, fasting serum non-esterified fatty acids (NEFA) and maximal aerobic capacity, measured as VO_2_max normalized to kg LBM, were not significantly different between groups. Consistent with our previous study in of the cohort from which these subjects were recruited [20], there were no significant differences in serum lipids or other circulating markers of cardiovascular risk between subjects segregated for insulin sensitivity (data not shown).

**Table 1:** Clinical characteristics.

	Insulin Sensitive(N = 10)	Insulin Resistant(N = 10)
**M/I** (mg^−1^⋅min^−1^⋅kg_LBM_^−1^⋅SSI^−1^)	15.4±0.6	7.5±0.6*
**ISI**	16.6±3.2	8.0±1.7*
**Sex** (male/female)	5/5	3/7
**Age** (years)	36±1	34±1*
**BMI** (kg/m^2^)	22.6±1.0	24.3±0.6
**VO_2_max** (ml/min/kg_LBM_)	58.1±1.5	53.2±2.9
**Serum NEFA** (µmol/L)	530±108	546±51
**Family History of T2D**	1	3

Values shown are mean ±S.E.M.

*P<0.05, RES *vs.* SEN groups.

### Skeletal Muscle Stress Kinase Activation

**Figure 1:**
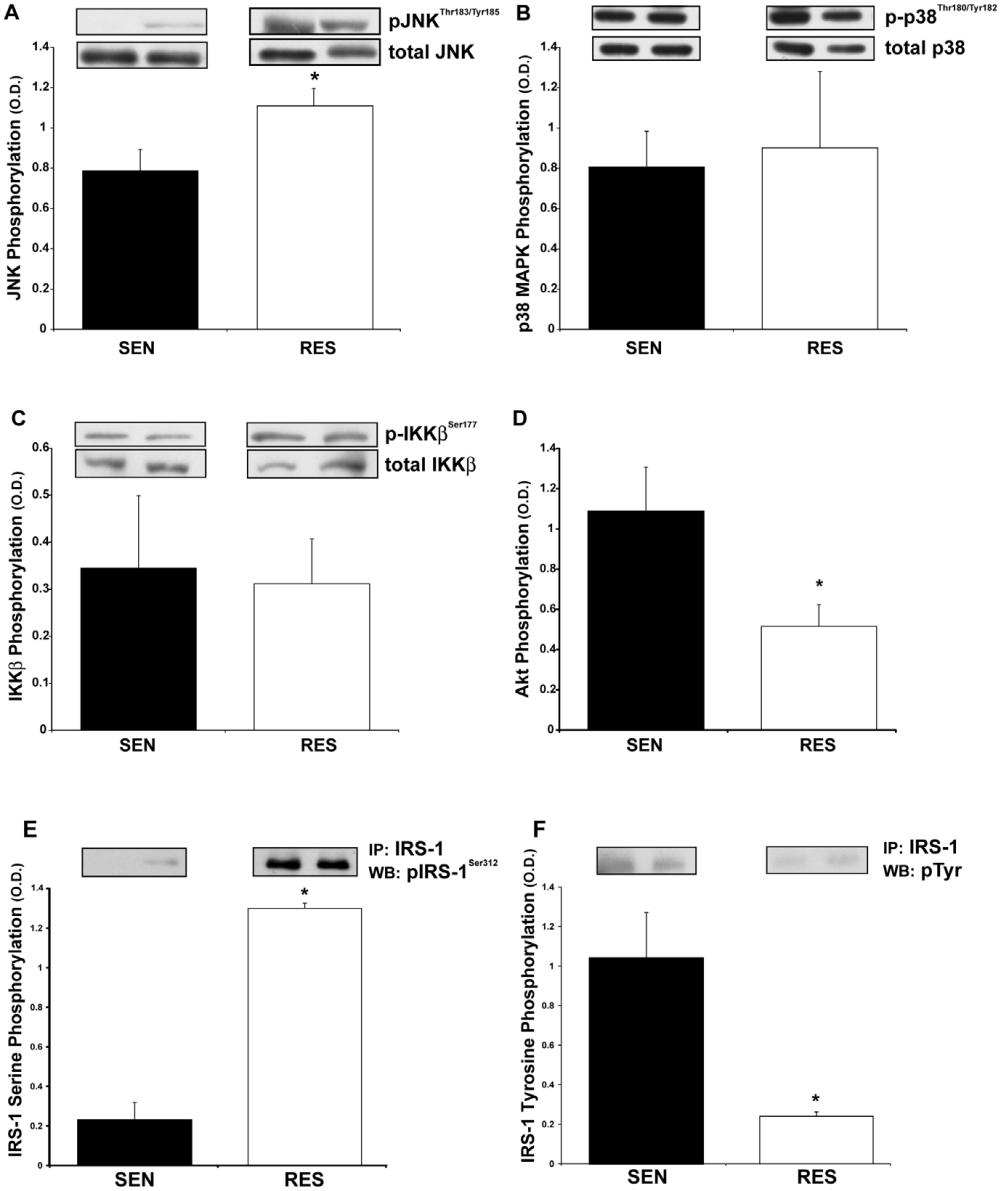
JNK activation in muscle from insulin resistant, non-obese subjects accompanies reduced insulin signaling and enhanced phosphorylation of IRS-1 on Serine^312^. Solubilized muscle biopsies obtained prior to insulin infusion were subject to SDS-PAGE, followed by Western blotting with phospho-specific antibodies against JNK Thr^183^/Tyr^185^ (**A**), p38 MAPK Thr^180^/Tyr^182^ (**B**), and IKKβ Ser^177^ (**C**). The predominant band (55 kD) recognized by the anti-JNK antibody is shown and was used for quantification. Optical density values were normalized to the content values for each protein as determined by Western blots for JNK, p38 MAPK and IKKβ, and are presented as mean ± SEM of the ratios of phospho- to total protein. *P<0.05 RES *vs.* SEN groups. (**D**) Muscle biopsies obtained after 2 hours of insulin infusion were solubilized and assayed for phosphorylation state of key second messengers of insulin signal transduction. Phosphorylation of Akt on Thr^308^ was determined by specific ELISA and normalized to total Akt content, also determined by ELISA. Values shown are mean ± SEM. *P<0.05, RES *vs.* SEN groups. Lysate volumes from insulin-stimulated muscle biopsies (4 SEN, 3 RES subjects) containing equivalent amounts of IRS-1 protein (as determined by ELISA) were immunoprecipitated with an antibody against IRS-1. Following SDS-PAGE, Western blotting was performed with a phospho-specific antibody against IRS-1 Ser^312^ (**E**), or with an anti-phosphotyrosine antibody (**F**). Blots were quantified by scanning densitometry and results expressed as mean ± SEM. *P<0.0005, RES *vs.* SEN groups.

Stress kinases previously demonstrated to negatively influence insulin signaling in obesity were studied for their relationship to insulin action in the non-obese population. We assessed the phosphorylation state of key enzymes involved in the JNK, NF-κB, and p38MAPK pathways in muscle biopsy samples obtained at the time of the glucose clamp procedure prior to insulin infusion. Activation of the JNK pathway was assessed by quantifying phosphorylation of JNK proteins on Thr^183^/Tyr^185^ by Western blot and normalizing these values to total JNK content. Phosphorylation of the 55 kD JNK isoform was significantly increased in muscle biopsies from the RES subjects (**Figure 1A**), while the 46 kD band, was not consistently observable, and therefore not quantified. Conversely, there was no significant difference in activation of the related kinase p38 MAPK between the RES and SEN groups, as assessed by phosphorylation of the regulatory Thr^180^/Tyr^182^ residues normalized to total p38 MAPK (**Figure 1B**). There were also no significant differences between groups in the total content of either JNK or p38 MAPK stress kinases (data not shown).

In order to assess activation of the NF-κB system, we measured both the phosphorylation of the activating serine kinase IKKβ and the content of the regulatory subunit IκB that is degraded following phosphorylation by IKKβ. There were no differences in either IKKβ phosphorylation or content (**Figure 1C**) between SEN and RES groups. The content of IκB was slightly, but not statistically, lower in the RES group (0.70±0.07 *vs.* 1.02±0.14 O.D. units, RES vs. SEN, respectively, *P* = 0.07). In the RES subjects, however, there were also significant reductions in the total content of both NF-κB p50 (0.51±0.04 *vs.* 0.78±0.10 O.D. units, RES vs. SEN, respectively, *P*<0.05) and p65 (0.22±0.06 *vs.* 0.46±0.09 O.D. units, RES vs. SEN, respectively, *P*<0.05) subunits. These observations suggest a general reduction in the protein components of the NF-κB system in muscle from the RES subjects, rather than an increased IKKβ-mediated degradation of IκB that would release NF-κB p50 and p65 subunits for nuclear translocation and subsequent activation of transcription.

### Skeletal Muscle Insulin Signaling

In order to determine if impairments in the insulin signal transduction pathway consistent with JNK mediated serine phosphorylation of IRS-1 were present in muscle from RES subjects, we studied insulin signaling in muscle biopsy samples under insulin-stimulated conditions. In these biopsies, obtained after insulin infusion during the glucose clamp procedure, we quantified IRS-1 serine and tyrosine phosphorylation and as well as the activation state of insulin signaling intermediates upstream and downstream of IRS-1.

We assessed both muscle IR content and IR tyrosine phosphorylation by ELISA. There was a trend toward reduced IR content in RES subjects that did not reach statistical significance (6.3±0.9 vs. 10.1±1.8 ng/mg, *P* = 0.07). However, tyrosine phosphorylation per IR was not different between groups (0.20±.03 vs. 0.15±0.02 O.D. units, RES vs. SEN, respectively, *P* = N.S.), consistent with impaired insulin action resulting from post-receptor mechanisms.

Downstream of the IR, however, reduced activation of insulin signaling intermediates were observed in the RES groups. Serine phosphorylation of Akt, a specific mediator of mediating insulin-stimulated glucose uptake, on the regulatory Thr^308^ residue was assessed by ELISA as a marker for Akt enzyme activation. We observed that insulin-stimulated phosphorylation of Akt was significantly lower in muscle biopsies from the RES compared to SEN subjects (**Figure 1D**).

Due to the requirements for substantial amounts of muscle protein for the immunoprecipitation procedures, we were able to assess IRS-1 phosphorylation state in only a subset of samples (4 SEN, 3 RES) for which adequate muscle protein was available. In these 7 samples (4 SEN, 3 RES), immunopurified IRS-1 was obtained and assessed for tyrosine and serine phosphorylation state by Western blot to assess whether elevated JNK activity in muscle from the RES group would be associated with corresponding alterations in IRS-1 phosphorylation state. In biopsies obtained following the 2 hour insulin infusion, phosphorylation of IRS-1 on SER^312^ was significantly increased in the resistant subjects (**Figure 1E**). This increase in serine phosphorylation was accompanied by a dramatic decrease in tyrosine phosphorylation of IRS-1 (**Figure 1F**). Total IRS-1 protein content, as measured by ELISA, was not different between SEN and RES groups (109±14 vs. 93±20 O.D. units, RES vs. SEN, respectively, *P* = N.S.).

### Intramyocellular Lipid Stores

**Figure 2:**
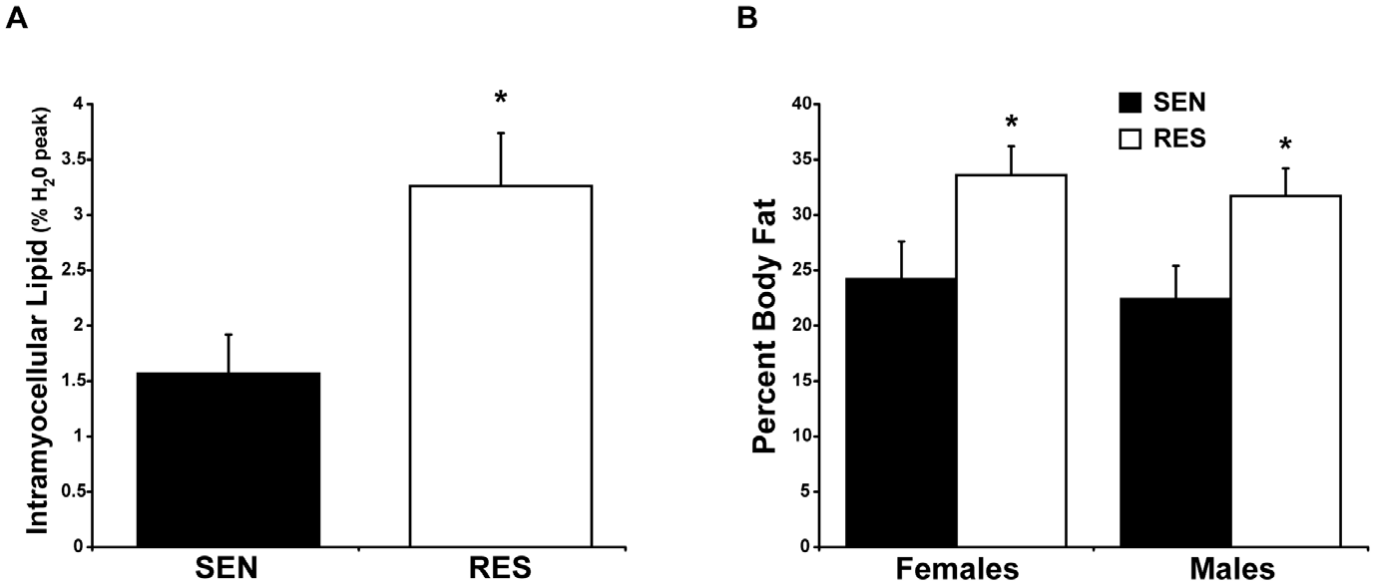
Intramyocellular lipid content and body fat stores are increased in insulin resistant, non-obese subjects. IMCL content of soleus muscles determined by 3D ^1^H-MRS multiple voxel technique and expressed as percent H_2_O peak (**A**). Values for percent body fat as determined by DXA are plotted separately males and females to show the independence of gender on the relationship between adipose stores and insulin action (**B**). Insulin sensitive (black bars) and resistant (white bars) groups are segregated based on results from hyperinsulinemic, euglycemic clamp as described. Values shown are mean ± SEM. *P<0.05, RES *vs.* SEN groups.

Results from *in vitro* studies, and studies of obese animals, indicate that activation of JNK and other stress kinases can result from either local lipid deposition within insulin target tissues, or as a result of circulating cytokines, such as TNF-α, that are released in greater quantities from expanded adipose depots. In order to determine if increased intramuscular lipid deposition or expanded whole body or abdominal adipose depots might be possible contributors to the increased muscle JNK activity, we first quantified IMCL by ^1^H-MRS in soleus and tibialis anterior (TA) muscles. IMCL content was two-fold higher in RES than SEN groups in both soleus (**Figure 2A**) and TA (0.78±0.10 *vs.* 0.40±0.07, respectively, *P*<0.01).

### Total and Abdominal Fat Stores

Although there were no significant differences in BMI between groups, we assessed whether total or abdominal fat stores might be increased in the RES subjects by DXA and MRI. We observed that body adipose stores were significantly increased in the RES compared to the SEN group. DXA analysis revealed that the RES group had higher percentages of total body fat (33.0±1.9 vs. 23.3±2.2 percent, *P*<0.05) and truncal fat (33.1±2.5 vs. 24.7±2.7 percent, *P*<0.05). Because body fat stores vary significantly between men and women, we examined the differences in body fat across the SEN and RES groups for men and women separately. Body fat stores varied more as a function of insulin sensitivity than gender (**Figure 2B**). Thus, the data regarding the association between adiposity and insulin action in the present study were not influenced by a higher percentage of females comprising the insulin resistant group.

We then quantified abdominal fat deposits both subcutaneously and within the abdominal cavity by MRI. MR images sufficient for precise discrimination of fat depots were obtained for all RES subjects, but for only 8 of 10 SEN subjects (2 males, 6 females). Consistent with the data obtained by DXA, MRI derived values for total abdominal fat tended to be higher in RES subjects. Total subcutaneous adipose tissue (SAT) content of the abdomen was 50% greater in the RES than the SEN group (252±34 *vs.* 170±25 cm^2^), although these differences failed to reach statistical significance (*P* = 0.07). There was no difference in the relative proportion of total subcutaneous adipose tissue comprising the deep subcutaneous (DSAT) rather than the superficial subcutaneous (SSAT) abdominal compartments (DSAT/SAT ratio = 0.50±0.02 *vs.* 0.50±0.03, respectively, *P* = N.S.). The amount of visceral adipose tissue (VAT) was not significantly different between the SEN and RES groups as a whole (48.5±4.0 vs. 64.8±11.4 cm^2^, SEN *vs.* RES, respectively, *P* = 0.20). The modest, non-significant differences between groups was attributable entirely to higher VAT content in RES men vs. SEN men (54.8±5.6 *vs.* 114.3±0.7 cm^2^, SEN *vs.* RES, respectively, *P*<0.05), while there was no difference in VAT between SEN women and RES women (42.3±3.9 *vs.* 43.6±5.4 cm^2^, SEN *vs.* RES, respectively, *P*<0.05).

### Serum TNF-α

We assessed the possibility that the expanded fat depots in RES subjects resulted in an increase in inflammatory cytokines as is observed in obese individuals, by measuring circulating levels of TNF-α. Serum TNF-α was correlated with both total fat mass (r = 0.466, P<0.05) and VAT (r = 0.564, P<0.05). Multiple regression analysis indicated that VAT was a stronger correlate of serum TNF-α, although the relationship between VAT and TNF-α independent of fat mass did not reach statistical significance (P = 0.07). However, TNF-α levels were not significantly greater in the RES than the SEN group overall (1.56±0.24 *vs.* 1.02±0.16 pg/ml, respectively, *P* = 0.09). While neither variable was related to insulin sensitivity across groups, circulating TNF-α levels across all subjects were significantly correlated with the phosphorylation state of IKKβ (r = 0.51, *P*<0.05).

### Skeletal Muscle Mitochondrial Content

We examined the possibility that the increased IMCL content in the RES group was associated with decreased muscle oxidative capacity by employing a Western blot for the enzyme succinate dehydrogenase (SDH) as a marker for muscle mitochondrial content. There was no difference in skeletal muscle SDH content between SEN and RES groups (1.59±0.10 *vs.* 1.59±0.07 OD units, SEN *vs.* RES, respectively, *P* = N.S.).

## Discussion

In the present study, we observed that insulin resistance in healthy, non-obese, non-diabetic subjects was accompanied by an increased activation of the JNK pathway with a corresponding increase in IRS-1 serine phosphorylation in skeletal muscle tissue, the site responsible for the majority of insulin-mediated glucose disposal [3]. These indices of stress kinase activation were accompanied by reduced post-receptor activation of the insulin signaling pathway at the level of IRS-1 and Akt. Consistent with a possible causal role of lipids in JNK activation, we observed increased levels of intramyocellular lipids, and higher total and abdominal adipose stores in the insulin resistant subjects, despite their normal body weight.

Our principal findings were activation of JNK and dramatic increase in phosphorylation of IRS-1 on SER^312^ in the insulin resistant non-obese subjects. JNK has previously been implicated only in obesity-mediated insulin resistance [21]–[23]. Increased serine phosphorylation of IRS-1 has been demonstrated in insulin resistant, non-obese subjects previously, but only in a carefully selected population of first degree offspring of diabetic parents [15]. In those studies, however, no specific serine kinase was implicated. While our data do not allow us to rule out the contribution of other cellular serine kinases on the altered phosphorylation state of muscle IRS-1, it is highly plausible that JNK can serve as a causal mechanism for insulin resistance in this population.

We observed that the intracellular triglyceride content of soleus and tibialis anterior muscles were twice as high in the insulin resistant group in our study, indicating the potential for lipotoxic effects in muscle cells of these subjects. When this condition is modeled in cultured hepatocytes by incubation with saturated fatty acids, a direct activation of the JNK pathway and serine phosphorylation of IRS-1 results [24], [25]. JNK activation in this model is directly attributable, at least in part, to oxidative stress resulting from increased lipid oxidation [25]. Thus, a large component of the toxic impact of cellular lipids may occur as a result of the burden of high rates of lipid oxidation on mitochondria, due to the increased generation of reactive oxygen species [26], which, in addition to activating JNK, may also impact insulin signaling via the p38MAPK and NF-κB pathways [27]. We could not, however, find any evidence for p38MAPK or NF-κB activation in the present study. The influence of oxidative stress on insulin signaling is likely complex, however, and is not yet fully understood. It is known that angiotensin II can both contribute to oxidative stress-induced insulin resistance [28], and directly result in serine phosphorylation of IRS-1 [29], but the specific kinases involved and the role of this hormone on human models of insulin resistance have yet to be determined. Beyond oxidative stress, high rates of lipid oxidation can also lead to incomplete lipid oxidation, with a resultant accumulation of β-oxidation intermediates [30]. These β-oxidation intermediates, such as acyl-CoAs or acyl-carnitines, have been linked to insulin resistance [30], [31], although their potential impact on insulin signaling is unclear.

It was not possible to determine the amount of lipid oxidation occurring in muscle, or to assess markers for oxidative or ER stress in the present study, although assessing these parameters along with additional components of JNK signaling will be important avenues for subsequent studies. Therefore, we can not conclude whether serine kinase activation is associated with the process of metabolizing excess lipids, or as a result of a direct effect of intracellular lipids intermediates on stress kinase pathways. Protein kinase C (PKC) enzymes, for example, can be directly activated by lipid intermediates such as long chain fatty acyl-CoAs, diacylglycerols, and ceramides [32] are associated with insulin resistance in humans [33]. While the MRS technique we employed quantifies the relatively benign triglyceride component of the intracellular lipid pool, IMCL measures have been used as surrogate indices of these intermediates. Due to constraints in sample size, we were unable to determine the amount of distinct lipid intermediate species or to assess activity of PKC enzymes. Further work will be needed to explore the potential role for PKC activation in this population.

It can not be ruled out that JNK activation in this population also results from the effects of increased lipid stores in adipose depots throughout the body. JNK can be activated via receptors for adipose-derived cytokines such as TNF-α and IL-1 [25], [34], and cytokines such as these have been implicated in translating the negative effects of expanded adipose stores into impaired insulin signaling in muscle. Insulin resistant subjects in the present study had significantly increased total fat stores and greater fat volume in the abdominal region. Still, the total amount of adipose tissue in these subjects is significantly less than that observed in obese subjects, making it unclear whether a negative impact of adiposity on muscle insulin action can be observed at this range of adiposity. There are little data to suggest that potential adipokine mediators of muscle insulin resistance are increased in non-obese subjects. In the present study we found some evidence that, even in this absence of obesity, increased fat stores have the potential to produce a detrimental effect on muscle insulin action.

Circulating levels of TNF-α are increased in obese states. Although the difference in serum TNF-α between the RES and SEN groups did not reach statistical significance, serum TNF-α was correlated both with total fat and visceral fat across subjects. While TNF-α can produce activation of the JNK pathway, it has been demonstrated that disruption of insulin signaling in cultured muscle cells by TNF-α is mediated by p38 MAPK [35]. Neither activation of p38 MAPK, nor the TNF-α sensitive IKKβ were observed in our resistant subjects when compared to the sensitive subjects. Thus, circulating TNF-α is not likely to constitute a primary determinant of JNK activity and insulin signaling in this non-obese population. The correlations between TNF-α and adiposity, as well as muscle IKKβ, suggest that even in non-obese subjects, small increases in total and visceral fat mass, although relatively small compared to values for obese subjects, are still sufficient to produce increased circulating levels of TNF-α. The lack of an association between these variables and insulin action suggest that a threshold may be required for TNF-α to activate the NF-κB and other pathways sufficiently to negatively influence insulin signaling, and that this threshold is not readily met in non-obese subjects.

The roles of other potential adipose-derived cytokines in this population remain to be determined, and can not be excluded as contributors to JNK activation or insulin resistance in this population. Likewise, non-adipose derived inflammatory factors could contribute to muscle stress kinase activation. Homocysteine has been identified as an activator of JNK [36]. However, homocysteine levels were not different between these groups, and have been shown to not associate with insulin resistance in this non-obese population [20].

While the causes of JNK activation and impaired insulin signaling in this population remain uncertain, it is clear that the insulin resistant subjects in our study represent a less dramatic version of an obese phenotype with an overabundance of adipose and muscle lipid storage, and elevated muscle JNK activation. An obese phenotype in non-obese subjects with metabolic disturbances has been described previously [16], [37]. These data, as with our data on serum lipids and cardiovascular risk in this same population [20], suggests that the threshold at which lipid deposition may impact metabolic health may be relatively low, and well within the range of what would be considered healthy weights. Our finding of increased IMCL levels in the insulin resistant subjects is also is in agreement with other reports demonstrating in association of increased IMCL levels with insulin resistance in non-obese populations [17]–[19], [38], [39], suggesting that this local accumulation of lipids in muscle is an important component of the development of insulin resistance in the absence of overall obesity.

It has been suggested, based on studies of first degree offspring of diabetic patients, that IMCL accumulation occurs in lean subjects as a result of impaired mitochondrial content and capacity [15], [40]. In contrast to studies in that unique population [15], we found no evidence for a reduced mitochondrial content as assessed by SDH, suggesting a substantial difference between these populations. While SDH content is a very crude estimate of mitochondrial content, the variables employed in the present study to measure total, abdominal and visceral fat, as well as IMCL values, were all correlated across subjects, as has previously been observed for subjects with lower total fat stores [17]. Our data is therefore suggestive of an accumulation of fat across multiple depots and tissue beds driving a phenotype similar to obesity, rather than a mechanism promoting tissue-specific lipid deposition, that results in insulin resistance. Whether the accumulation of lipids that does occur in muscle provides a disproportionate negative impact on insulin signaling in cells of that tissue, as compared to other fat stores, remains to be determined. Further studies are required to determine whether reduced mitochondrial content is indeed a significant contributor to muscle lipid accumulation and insulin resistance, and whether the first degree offspring of diabetic parents present with a unique phenotype across the spectrum of insulin resistance. The present study included a subgroup of subjects with a parental history of diabetes. While the possibility exists that this introduced heterogeneity into the determinants of insulin resistance, our study was designed to assess mechanisms of insulin resistance in the general population, which includes subjects both with and without the potential for genetic influences on insulin resistance.

Although some studies of non-obese subjects have employed a BMI of 25 as the cutoff point for normal weight, our results were not influenced by the inclusion of subjects with BMI values up to 27. When only the data from those individuals with BMI values ≤25 were analyzed, the results were identical; insulin resistance was associated with increased JNK activation reduced insulin signaling, and elevated intramyocellular lipids, abdominal and visceral fat. Likewise, there was no difference in the trends of the data when men and women were analyzed separately, except for the trend for insulin resistant men to have higher amounts of visceral fat than insulin sensitive men, a trend not observed for women. However, the sample size involved precludes drawing any conclusions from these data, and further studies would be required to assess any gender differences in visceral fat accumulation, or the potential for visceral fat depots to influence insulin action in this population.

The group analysis aspect of the study design facilitated the identification of characteristics that differentiated insulin resistant from insulin sensitive subjects in an otherwise healthy population. Pre-screening for insulin-mediated glucose disposal with subsequent enrollment of only the most insulin sensitive and resistant subjects allowed for in depth study of a sufficient number of subjects with substantial insulin resistance, considering that there is no accepted clinical threshold to identify insulin resistance. This design did not, however, allow us to run uni- and multivariate analyses in an attempt to identify independent determinants of insulin resistance of JNK activation. Further cross sectional studies are needed to segregate the various contributors to JNK activation and insulin resistance in this population.

In summary, we observed that insulin resistance in the non-obese population is associated with an activation of the JNK pathway with increased serine phosphorylation of IRS-1. Implicated in this disruption of cellular insulin action is the accumulation of lipids within skeletal muscle, and the greater degree of overall adiposity that was observed in the insulin resistant subjects. Additional work is required to study additional stress kinase pathways in greater depth, and to assess the extent that these factors individually contribute to insulin resistance across the non-obese population.

## Materials and Methods

### Subjects

Non-obese, healthy subjects between the ages of 20 and 50 were recruited from the local population. A body mass index (BMI) cutoff of less than 27 (25 for Asian Americans [41]) was chosen due to the wide range of insulin sensitivity values with no correlation to BMI reported for this population [42]. Women were premenopausal. Individuals with diabetes, cardiovascular diseases, HIV and other active infections, thyroid disorders, epilepsy, cancer, hepatitis, cystic fibrosis, sickle cell disease, asthma or renal disease were excluded. Subjects taking any medications known to affect insulin sensitivity, carbohydrate metabolism, or lipid metabolism were excluded. Exercise and general physical activity pattern were determined using the questionnaire developed by Baecke et al [43]. This questionnaire generates a physical-activity index score from 3 to 15 based on work, sport and leisure time energy expenditure, with each category scored from 1 to 5 (lowest to highest activity level). Subjects with scores greater than 10 (population mean approximately 8.3) were not enrolled in the study.

### Ethical considerations

All subjects gave informed, written consent. The protocols and consent forms were approved by the University of California, San Francisco institutional review board and Clinical Research Center where the study was conducted.

### Identification of insulin sensitive and insulin resistant subjects

The subjects participating in this study were part of a larger cohort of subjects meeting the above entry criteria that were employed in a study of the impact of adiposity and insulin resistance on serum lipids and cardiovascular risk in the non-obese population [20]. As such, data on multiple aspects of cardiometabolic health in the population a larger group of non-obese individuals that included these subjects has been presented elsewhere. As a result of excluding individuals with BMI>27 and those with impaired glucose tolerance or fasting hyperglycemia, the pool of subjects did not include any individuals meeting the criteria for the metabolic syndrome.

Insulin sensitivity index (ISI) values determined as part of the larger study were employed as the first of a two-step screening process to identify insulin sensitive and resistant subjects for enrollment in the present study. ISI values were calculated from a 75 g oral glucose tolerance test (OGTT) according to the formula by Matsuda and DeFronzo [ISI = 10,000/√(fasting glucose × fasting insulin) × (mean glucose × mean insulin)] [44]. Subjects with impaired glucose tolerance or impaired fasting glucose were excluded.

Qualifying subjects with high or low ISI values (greater than 13 or less than 9) were invited to participate in further screening by hyperinsulinemic euglycemic clamp [45]. Glucose disposal values (M/I) were calculated as mg glucose infused per min per kg lean body mass divided by steady state insulin levels (in µU/ml×100) between 90 and 120 minutes of the 2 hr clamp (insulin infusion rate 80 mU/m^2^⋅min body surface area). M/I values from 39 subjects were divided into tertiles, and the values dividing the middle tertile from the lower and upper tertiles were taken as cut points to determine eligibility for enrollment in insulin resistant (<9.5) and insulin sensitive (>12.0) groups for subsequent studies. Groups were not matched for sex, but subsequent separate analyses of group differences across sexes indicated that, except where noted, the presence of more females in the insulin resistant group (7 of 10) than the sensitive group (5 of 10) did not influence the overall group differences.

### Blood Chemistry

Glucose was determined in whole blood by the glucose oxidase technique (Sigma). Insulin levels were measured by ELISA (Millipore, Billerica, MA). Serum non-esterified fatty acids were measured using Wako Diagnostics kit (Richmond Virginia) and serum TNF-α were measured using the Quantikine HS high sensitivity ELISA (R & D Systems, Minneapolis, MN) following manufactures instructions.

### Total and abdominal adipose tissue assessment

Body composition was assessed by dual-energy X-ray absorptiometry (DXA). Visceral (VAT) and subcutaneous (SAT) abdominal adipose tissue areas as well as deep subcutaneous abdominal adipose tissue (DSAT), and superficial subcutaneous abdominal adipose tissue (SSAT) were assessed by magnetic resonance imaging acquired with a GE Excite 3-T scanner as previously described [46]. Acquisition was prescribed from a sagittal scout so that the image plane passed through the center of the vertebral disc between vertebrae L4 and L5, and calculated using a customized software program written in the interactive data language platform (IDL Research Systems, Inc., Boulder, CO) [47].

### Intramyocellular lipids by ^1^H-MRS

As described previously [46], muscle spectral and image data were acquired from calf muscles using a quadrature knee coil. Single voxel Point Resolved Spectral Selection (PRESS) technique was used to obtain spectra from tibialis anterior (TA) muscle compartment due to size limitation of the muscle compartment, while a 3D multi voxel technique was used to obtain spectra from soleus muscle. Each slice was measured three times and the average value was taken as previously described [48].

### Muscle Biopsies

At time of the euglycemic hyperinsulinemic clamp, percutaneous muscle biopsies were obtained from the belly of the *vastus lateralis* both prior to and after 120 minutes of insulin infusion (prior to conclusion of the infusion protocol) on opposite legs. After local anesthesia, a 5 mm diameter Bergstrom needle was passed through a 7 mm skin incision and subcutaneous tissue, and then advanced approximately 2 cm beyond the muscle fascia. The biopsy (50–100 mg tissue) was obtained with applied suction. The incision was closed with steri-strips and firm pressure applied.

### Sample Preparation

Biopsy specimens (50–100 mg wet weight) were frozen in liquid nitrogen, pulverized and homogenized in 50 mM HEPES, pH 7.4, 1% Triton X-100, 100 mM NaF, 10 mM Na4P2O7, 1 mM PMSF, 2 mM Na3VO4, 2 µM leupeptin, 20 µg/ml aprotinin. Homogenates were centrifuged at 100,000×g for 60 minutes and lysates discarded. The pelleted samples were then solubilized for 1 hr at 4°C and then centrifuged at 100,000×g for 60 min at 4°C, and stored at −80°C. Protein was measured by the Bradford Assay (Pierce Chemicals).

### Immunoprecipitation/Immunoblotting

Content and phosphorylation of IR were measured by ELISA [49]. Content of IRS-1, total AKT/PKB and serine phosphorylated AKT/PKB were each measured by ELISA (Cell Signaling Technologies). Content and activation state of JNK, IKKβ, and p38 MAPK enzymes were determined by Western blots employing antibodies to measure total and phosphorylated proteins (Cell Signaling). Values for protein phosphorylation were normalized to total protein content to determine the activation state of individual kinases. Muscle GAPDH and SDH were also quantified by Western blot (AbCam Inc). To measure IRS-1 phosphorylation, identical amounts of IRS-1 for each sample tested were first immunoprecipitated by anti-IRS-1 antibody (Millipore Corp) and then subjected to Western blot analyses with either anti-phosphotyrosine (anti-PY99, Santa Cruz Biotechnologies) or anti-phosphoserine IRS-1 Serine^312^ (Millipore Corp) antibodies.

### Statistical analysis

Statistics were calculated using MedCalc statistical software (MedCalc Software, Mariakerke, Belgium). Differences between insulin sensitive and resistant groups were analyzed by Student's *t*-test. Correlations between variables were determined by Pearson correlation coefficients. Significance was set at *p*<0.05. All data are presented as mean ± SEM.
